# Plant-Derived Nutraceuticals and Immune System Modulation: An Evidence-Based Overview

**DOI:** 10.3390/vaccines8030468

**Published:** 2020-08-22

**Authors:** Antonella Di Sotto, Annabella Vitalone, Silvia Di Giacomo

**Affiliations:** Department of Physiology and Pharmacology, Sapienza University of Rome, P.le Aldo Moro 5, 00185 Rome, Italy; silvia.digiacomo@uniroma1.it

**Keywords:** immune system boosters, immunosuppressors, polysaccharides, fatty acids, labdane diterpenes, punicic acid, oleic acid, γ-linolenic acid, β-glucans, andrographolide

## Abstract

Immunomodulators are agents able to affect the immune system, by boosting the immune defences to improve the body reaction against infectious or exogenous injuries, or suppressing the abnormal immune response occurring in immune disorders. Moreover, immunoadjuvants can support immune system acting on nonimmune targets, thus improving the immune response. The modulation of inflammatory pathways and microbiome can also contribute to control the immune function. Some plant-based nutraceuticals have been studied as possible immunomodulating agents due to their multiple and pleiotropic effects. Being usually more tolerable than pharmacological treatments, their adjuvant contribution is approached as a desirable nutraceutical strategy. In the present review, the up to date knowledge about the immunomodulating properties of polysaccharides, fatty acids and labdane diterpenes have been analyzed, in order to give scientific basic and clinical evidence to support their practical use. Since promising evidence in preclinical studies, limited and sometimes confusing results have been highlighted in clinical trials, likely due to low methodological quality and lacking standardization. More investigations of high quality and specificity are required to describe in depth the usefulness of these plant-derived nutraceuticals in the immune system modulation, for health promoting and disease preventing purposes.

## 1. Introduction

Immunomodulators are defined as agents able to affect the immune response, which represents the set of reactions activated to protect the organism against infective agents, environmental injuries and illness; moreover, immune response can counteract the invasion of harmful native cells, such as precancerous and cancerous ones [1].

**Figure 1:**
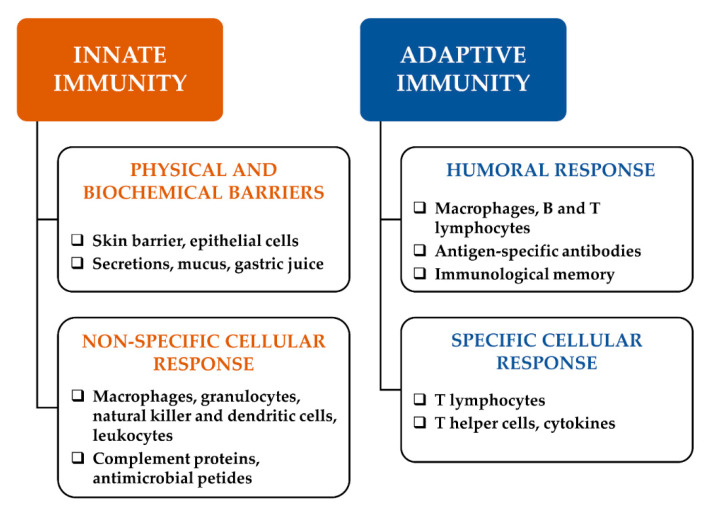
Responses involved in innate and adaptive immunity. Fast and nonspecific responses, occurring against all factor identified as nonself, are involved in innate immunity; conversely, adaptive immunity is a highly specific, complex and slow response mediated by T and B lymphocytes, which release antigen-specific antibodies and cytokines. Immunomodulators can directly affect innate and adaptive response or the factors involved, thus leading to immunostimulant or immunosuppressive effects.

Immune response is mediated by a first line of defence, namely innate immunity (Figure 1), which is characterized by physical and biochemical barriers, alongside a non-specific cell-mediated immune response, including granulocytes, macrophages, natural killer cells and humoral elements, which cooperate to counteract pathogen infection and malignant transformation [2]. Moreover, an adaptive immunity is activated as a second defense line after a macrophage-mediated presentation of antigens to B lymphocytes, with the help of T lymphocytes; then, B cells can mediate the humoral immunity through the production of high-affinity antibodies and establish immunological memory [3]. Moreover, T lymphocytes can mediate cellular immunity after activation by cytokines released from helper T cells [2].

Immunomodulating agents can affect immunity in a negative or positive manner, thus being categorized as suppressing or stimulant [4]. Particularly, immunosuppressors inhibit the activation of immune response or decrease the activity of its components, thus restoring normalcy. They are of interest in organ transplantations and in autoimmune disorders, wherein the immune system mistakenly activates an immune response against the own body tissues, leading to their destruction [4]. For instance, vitamin D has been shown to counteract the aberrant immune responses of systemic lupus erythematosus, without compromising the physiological defences and to produce benefits in atopic dermatitis too [5,6].

Conversely, immunostimulants boost the endogenous immune defences, thus allowing one to restore or maintain the body homeostasis [4]. They can be usefully exploited as immunotherapeutic agents by individuals with immunocompromised conditions; however, they can represent suitable prophylactic strategies for healthy individuals or more susceptible subjects against viral infections [4]. In support, during the current SARS-CoV-2 pandemic, the trained immunity by vaccines, which induce heterologous protection, have been proposed as a rational strategy to boost antiviral defences and reduce susceptibility to infection [7].

A further group of immunomodulators is represented by immunoadjuvants, able to enhance immune response to vaccines without producing specific antigenic effects, and more recently approached as adjuvant pharmacological treatments, especially for viral infections and cancers [8,9,10,11,12].

Immune-associated disorders, including autoimmune diseases, viral or bacterial infections, and chronic diseases, are usually associated with acute inflammation, which represents a key component for the activation of immune response [13]. On the other hand, the chronicity of inflammatory response can negatively influence the immune function, affecting both innate immune cells and T and B lymphocytes, thus suggesting a possible usefulness of anti-inflammatory immunomodulators [13]. Accordingly, immunomodulatory agents, with antioxidant and anti-inflammatory activity, have attracted great attention as possible chemopreventive agents, due to their ability to counteract chronic inflammation, which provides favorable conditions for the transition from normal to cancer cell [14].

Furthermore, immunostimulants can act as adjuvant anticancer treatments, to counteract their immunosuppressive side-effects [14].

Growing evidence highlighted an important role of gut microbiome in the maintenance of immune function, and the disruption of the microbiome seems to have a role in disease development [15]. This intimate microbiome–immune system crosstalk suggests that boosting the resident microbiome, through pre/probiotic supplementations or suitable intervention for preventing disruption of microbial communities, can reinforce the immune defences, thus representing an alternative immunomodulatory strategy [16].

Several medicinal plants and phytochemicals are known since the ancient time for their ability to modulate the immune system function [17]. As major immunomodulatory mechanisms, they act especially as boosters of immune system, through the stimulation of both innate and adaptive humoral and cellular immunity (Table 1). However, further mechanisms, such as an interference with proinflammatory pathways and a modulation of the gut microbiome, have been reported [18,19,20].

The best known immunostimulant species is echinacea (*Echinacea purpurea* (L.) Moench, *E. pallida* Nutt. and *E. angustifolia* DC), characterized by immunostimulant properties on both innate and adaptive response, associated with antiviral, antinflammatory and antimicrobial effects [42]. More recently, its ability to indirectly boost the immune system, through the modulation of gut microbiome, has been highlighted [18]. All the phytocomplex seems to be involved in these properties, however, alkylamides and purified polysaccharides have been found to induce immunomodulatory effects [42,43]. The immune boosting properties of echinacea, particularly as enhancers of macrophage and lymphocyte activation, have also been highlighted in clinical studies; however, further high-quality studies are required for better characterizing the possible usefulness of echinacea byproducts as immunomodulators [44]. 

Several preclinical and clinical evidence also highlighted the ability of *Curcuma longa* L. to activate cellular immunity and to interfere with inflammation, thus acting as an immune booster [42]. 

Furthermore, some adaptogenic plants, such as *Panax ginseng* C.A. Meyer, *Astragalus membranaceous* (Fisch.) Bge., and *Withania somnifera* (L.) Dunal can indirectly stimulate the immune system, through improving the resistance to stress [35,45]. Indeed, ginseng has been reported able to prevent several diseases through modulating the immune system and gut microbiome, thus suggesting its usefulness as an immunity boosting dietary supplement [36]. Similarly, the immunomodulatory properties of *W. somnifera* and its constituent withaferin A have been reported [39]. At last, preliminary studies have highlighted the immunomodulatory effects of other medicinal plants, such as *Asparagus racemosus* Willd., *Azadirachta indica* A. Juss., *Artocarpus tonkinensis* A. Chev. Ex Gagnep, *Syzigium aromaticum* (L.) Merr. and L.M. Perry and *Hypoxis rooperi* T. Moore [26,33,37,46,47]. 

Details about the bioactive constituents and the mechanisms of actions of medicinal plants known to modulate the immune function are reported in Table 1.

**Figure 2:**
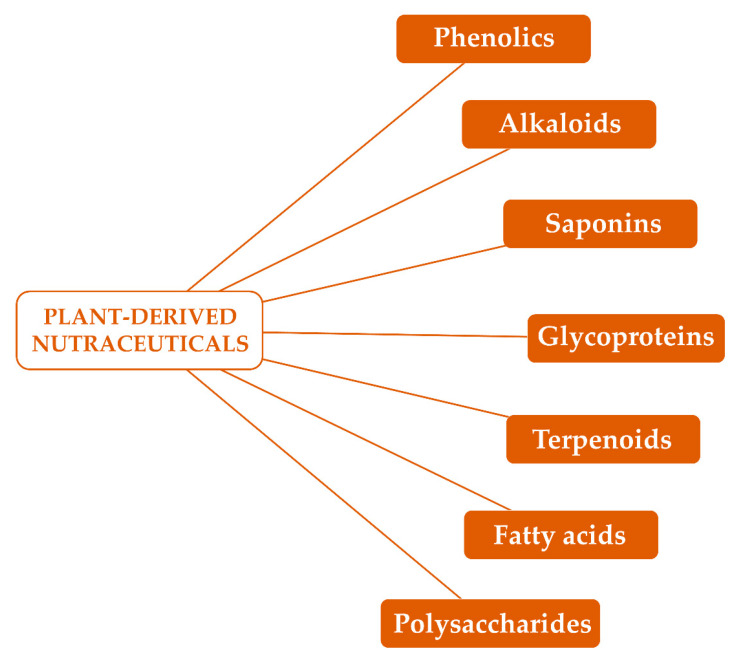
Major groups of plant-derived nutraceuticals able to modulate the immune function.

Several phytochemicals, especially alkaloids, phenolics, glycoproteins and saponins (Figure 2), have been reported to possess antinflammatory and immune modulating properties [48]. Moreover, terpenoids, polysaccharides and fatty acids (Figure 2) have received considerable attention as possible immunomodulatory agents for further application in immune disorders [48,49]. 

Particularly, aristolochic acid, an alkaloid from *Aristolochia clematitis* L., showed immunostimulatory properties, by enhancing the phagocytic activity of peritoneal macrophages and leukocytes; however, its potential cannot be exploited, because of its carcinogenic risk [50]. Likewise, vincristine and staurosporine act as immunostimulants at low doses, while as immunosuppressors at higher doses [51]. Among polyphenols, resveratrol stimulated both cellular and humoral immunity in preclinical models, thus preventing pathogen replication and inflammation, and promoted antitumor immune response too [52]. Furthermore, cichoric acid from echinacea promoted phagocytic activity, both *in vitro* and *in vivo* [53]. Anti-inflammatory and immune-modulatory effects has been highlighted for curcumin too, although the poor bioavailability limits its clinical application [54].

In the present review, up to date knowledge on the scientific basis for the immunomodulatory activity and clinical relevance of some emerging classes of plant-derived nutraceuticals, including polysaccharides, fatty acids and labdane diterpenes, has been reported. A comprehensive search was made using PubMed and SCOPUS electronic databases and selecting English as the preferred language, although no language limitations nor filters were applied. For more specific requirements, Google Scholar and ClinicalTrials.gov were considered too. The following searching keywords and their combinations through the Boolean logical operators were used: “herbal immunomodulators”, “phytochemicals”, “immune system”, “nutraceuticals”, “medicinal plants”, “immunomodulation”, “immune system boosters”, “immunosuppressors”, “immunoadjuvants”, “gut microbiome”, “natural occurrence”, “chemical features”, “preclinical studies”, “clinical trials”, “polysaccharides”, “echinacea”, “astragalus”, “β-glucan”, “fatty acids”, “PUFA”, “oleic acid”, “punicic acid”, “γ-linolenic acid”, “linoleic acid”, “evening primrose oil”, “borage oil”, “flaxseed oils”, “labdane diterpenes” and “andrographolide”.

This overview allows one to identify novel immune system modulators to be usefully exploited for health promoting and disease preventing purposes.

## 2. Polysaccharides

### 2.1. Chemical Features

Polysaccharides are carbohydrate macromolecules containing at least 10 monosaccharide units, joined by glycosidic linkages to form long-chain molecules, which can be both linear and highly branched. They are called homopolysaccharides when constituted of the same monosaccharide unit, while heteropolysaccharides if different units are present. Some of them are also referred to as dietary fibres, meaning that these macromolecules are neither digested nor absorbed in the human small intestine [55].

**Figure 3:**
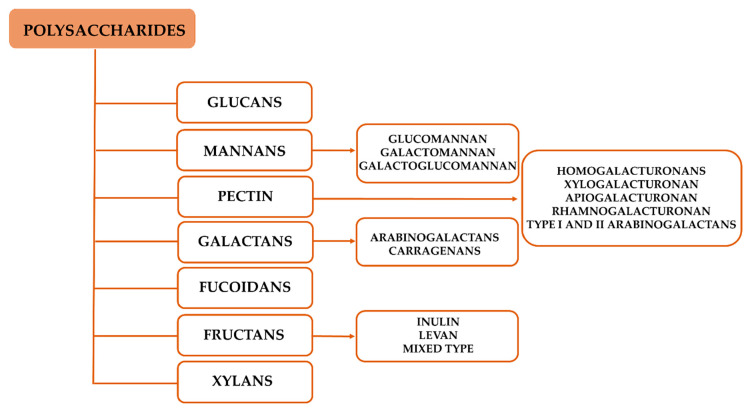
Schematic representantions of the different classes of polysaccharides associated with immune system modulation.

Several polysaccharides have been found to modulate both innate and adaptive immune responses, among which, glucans, mannans, pectins, fucoidans, galactans, fructans, and xylans are the most studied (Figure 3) [56].

**Figure 4:**
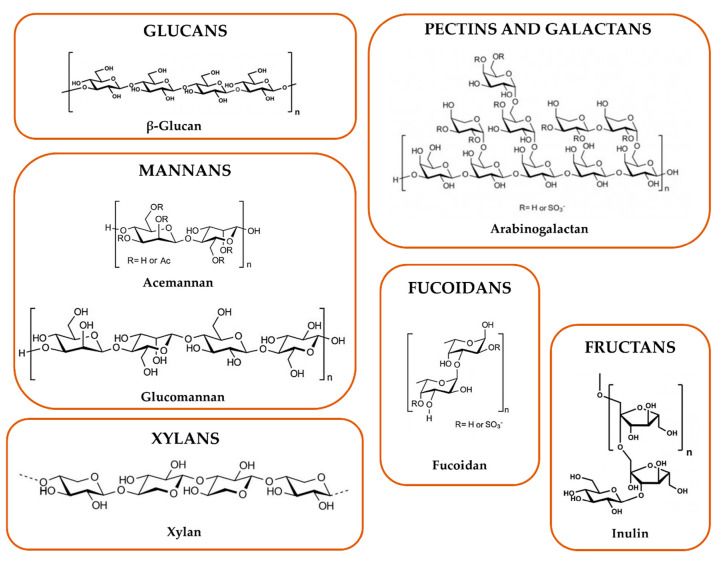
Chemical structure examples of the different classes of polysaccharides.

Chemical structure, molecular weight, conformation, the presence of functional groups (i.e., acetyl and sulfate groups), and branching have been identified as structural features for the immunostimulatory properties of polysaccharides. The chemical structures of the polysaccharides associated with immunomodulatory properties are displayed in Figure 4.

Glucans are based on the D-glucopyranosyl unit (homoglucans); the different glycosidic bonds, namely (β1→4), (β1→3), and (β 1→6) or (α1→3), (α 1→4), and (α 1→6), allow the production of linear and branched glucans. It seems that (β1→3)-D-glucan moiety, triple helix conformations, sulfation and carboxymethylation of (β 1→3)-D-glucans, and chain acetylation are involved in glucan immunostimulatory activity. Regarding (α1→6) (α1→4)-D-glucans, their structure activity relationship is less characterized [56].

Mannans consist of a D-mannose backbone, linked mainly by β1→4 bonds, which can be ramified with other monosaccharide, so originating glucomannan, galactomannan, and galactoglucomannan [57]. Furthermore, (β1→3)- or (α1→3)-, (β1→2)-, and (β1→6)- or (α1→6)-D-mannosidic bonds are reported [56]. The (β1→6)-D-mannan moiety (e.g., galctoglucomannans), acetyl and sulfate group presence, and this kind of branching seems to confer a high immunostimulatory activity [58,59,60].

Pectins are complex polysaccharides which contain a common galactopyranosyluronic acid. Homogalacturonans, xylogalacturonan, apiogalacturonan, rhamnogalacturonan, type I and II arabinogalactans belong to this class. Particularly, type I arabinogalactans (AG-I) possess an α-1-arabinofuranosyl and β-D-galactopyranosyl units linked via position 3 at the main chain, while type II arabinogalactans (AG-II) comprise highly branched polysaccharides with ramified chains of (β1→3)- and (β1→6)-D-galactopyranosyl units [61,62], to which the arabinosyl units might be attached. The degree of branching, methyl esterification, acetylation, and the type of branched chains and molecular weight determine the structural diversity [63]. Moreover, flexible chain conformation and branched regions are the main ones responsible for the immunomodulatory properties [64,65].

Galactans are polysaccharides rich in galactose and include, beside type I and II arabinogalactans, carrageenans, chemically characterized by repeating disaccharide units of sulfated or unsulfated D-galactose, that are linked by (β1→4)- and (α1→3)-bonds. Low molecular weight (<20 kDa) and a high degree of sulfation have been reported as features that high influence their immunomodulatory properties [66,67].

Fucoidans are heteropolysaccharides rich in L-fucopyranosyl sulfated units linked by (α1→2), (α1→3) or (α1→4) bonds. Other monosaccharides can be present, such as galactopyranosyl, mannopyranosyl, xylosepyranosyl and uronic acids [68]. The naturally higher content of sulfate groups and the presence of acetyl groups are associated with a higher stimulatory activity [56].

Fructans are polysaccharides which constitute up to 70 fructose units with a sucrolose terminal molecule. They are classified in inulin with a (β2→1)-D-fructofuranosyl, levan with a (β2→6)-D- fructofuranosyl, and mixed type, with both (β2→1)- and (β2→6)-linked d- fructofuranosyl moieties. A helical conformation has been associated with the modulatory activity on the immune system [69,70]. At last, xylans are polysaccharides containing predominantly a backbone of (β1→4)-D- xylosepyranosyl units. Other monomers attached to their backbone include α-D- glucopyranosyl A units (glucuronoxylans) and α-L-arabinofuranosyl units (arabinoxylans). A correlation between their structure and activity has not been elucidated yet [71,72].

### 2.2. Natural Occurrence

Polysaccharides are naturally occuring in animal body fluids, cell walls, bacteria, yeast and fungi, extra cellular fluids, and in plant seeds, stems and leaves, which represent the focus of the present review. The main advantage of plant polysaccharides seems to be the low toxicity with respect to immunomodulatory bacterial polysaccharides and synthetic compounds [73]. Thus, they represent an ideal alternative for immune modulation. A variety of polysaccharides with immunomodulatory properties have been discovered in different species of plants (Table 2). Among the most studied, there are type I and II arabinogalactans from *Astragalus membranaceus* (Fisch.) Bge., fructans from *Allium sativum* L. [56], fucogalactoxyloglucan and type II acidic arabinogalactan from *Echinacea purpurea* L. (Moench), ginsan and panaxanes from *Panax ginseng* C.A. Meyer, acemannan and aloeride from *Aloe vera* L. [74], and glucomannan from *Amorphallus konjac* Koch [75].

### 2.3. Pharmacological Properties: Preclinical Evidence

**Figure 5:**
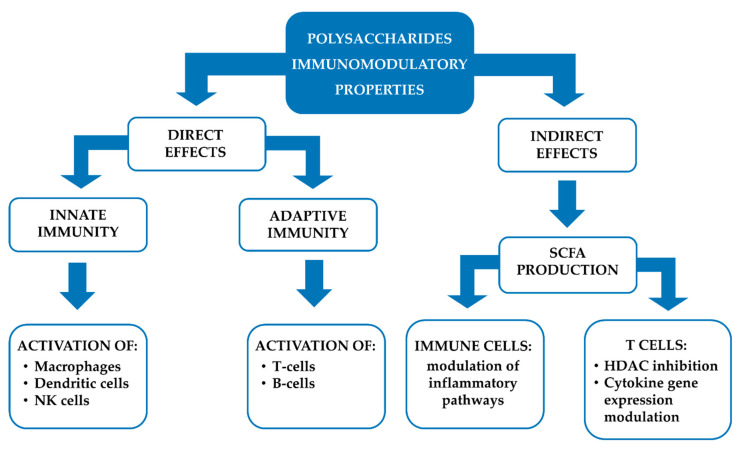
Schematic representantions of the direct and inderect effects of polysaccharides on the immune system. SCFA, short-chain fatty acids; NK, natural killer; HDAC, histone deacetylases.

Several studies have shown that polysaccharides from plants can modulate both innate and acquired intestinal immunity, by direct and indirect mechanisms. The former include the activation of immune cells (e.g., macrophages, dendritic cells, natural killer cells, T cells, B lymphocytes), while the latter the short-chain fatty acid (SCFA) formation (Figure 5).

The immunomodulatory effects of plant polysaccharides on macrophages are mainly achieved through the generation of reactive oxygen and nitrogen species (ROS and NOS), and the stimulation of cytokines secretion, cell proliferation, and macrophage phagocytic activity [101]. For example, *A. membranaceus* polysaccharides have been shown to promote nitric oxide (NO) synthesis in macrophages, by inducing the gene expression of inducible nitric oxide synthase (iNOS), through the activation of nuclear factor kappa-B (NF-κB)/Rel [102,103]. Moreover, they were also able to increase the macrophage phagocytic activity, by enhancing their secretion of release factor and intracellular Ca^2+^ concentration [104,105].

Pectic polysaccharides from *Citrus unshiu* Marc. have been shown to simultaneously regulate the expression of pro- and anti-inflammatory cytokines. Particularly, they increased the production of the pro-inflammatory cytokines tumor necrosis factor (TNF)-α and interleukin (IL)-6 and the anti-inflammatory cytokine IL-12 in macrophage RAW264.7, so showing a regulatory mechanism to maintain an equilibrium state [106]. Arabinogalactan from *E. purpurea* has been reported to increase macrophages activation and IL-1, TNF-α and interferon (IFN)-β production [86]. The activation of macrophages by plant polysaccharides seems to be due to specific receptors present on their surface, which initiates the immune response, and exerts an immunomodulatory effect. These receptors are called pattern recognition molecules, and include: Toll-like receptor 4 (TLR4), CD14, complement receptor 3 (CR3), scavenger receptor (SR), mannose receptor (MR), and Dectin-1. Their activation determines a series of intracellular signaling cascades, leading to the transcriptional activation and production of inflammation-related cytokines [101].

Immunity modulation by plant polysaccharides can be achieved also by modulating the cytokine release from intestinal dendritic cells. Indeed, pectin has been shown to reduce IL-6 and IL-10 release induced by the synthetic lipopeptide P3CSK4 [107]. Moreover, inulin, pectin, arabinoxylan and β-glucan have been found to elevate IL-10/IL-12 ratio and to reduce the release of IFN-γ, IL-12, IL-1, IL-6, IL-8, monocyte chemoattractant protein (MCP)-1, macrophage inflammatory proteins (MIP)-1α, RANTES and TNF-α by dendritic cells [108]. Polysaccharide enriched extracts of *E. purpurea* have been found to promote the phenotypic and functional maturation of dendritic cells by modulating c-Jun N-terminal kinase (JNK), p38 mitogen-activated protein kinase (MAPK) and NF-*κ*B pathways [109].

The activation of natural killer (NK) cells also contributes to the immunity modulation by polysaccharides. Indeed, it has been shown that *A. membranaceus* polysaccharides can enhance the activity and killing effects of NK cells and promote their proliferation in rats with gastric cancer [110]. Moreover, they were able to increase CD3-CD4-CD8+ NKs in peripheral blood lymphocytes [111]. NKs activation is probably due to the polysaccharides interaction with the killer cell lectin-like receptor K1(KLRK1) of NKs [112]. Arabinoxylans extracted from wheat bran have been shown to inhibit the growth of transplantable tumors, and to promote the NK cell activity in S180 tumor-bearing mice [113]. Moreover, the MGN-3 rice bran arabinoxylan showed to enhance natural killer (NK) cell activity in aged C57BL/6 and C3H mice upon its intraperitoneal injection [114].

Adaptive immunity is also modulated by plant polysaccharides. Particularly, Fan et al. have shown that polysaccharides from *A. membranaceus* significantly up-regulated the proliferation of B lymphocytes, probably through the interaction with immunoglobulin on the surface of B cells [115,116,117]. *A. membranaceus* polysaccharides were also able to increase the number of CD3+CD4+CD8+ memory T helper (Th) cells and CD3+CD4-CD8+ cytotoxic T cells [111]. Moreover, they also enhance the CD4+/CD8+ T cell ratio [118]. Furthermore, arabinoxylan were found to increase the activation of T- and B-cells and humoral and cell-mediated immunity in tumor bearing mice [71].

At last, β-glucan microparticles enhanced T-cell activation and proliferation *in vitro* [119]. Their ability to affect the immune system by inducing Th1 and/or Th2 type immune response makes polysaccharides suitable adjuvants of the vaccine. Among them, inulin, chitosan, glucans and mannans have been most extensively studied. Particularly, the gamma and delta forms of inulin fructan have shown adjuvant activity against infectious pathogens by stimulating both Th1 and Th2 responses without inducing immunoglobulin E (IgE) production [120]. Moreover, Advax, a polysaccharide derived from delta inulin, has demonstrated to increase the immunization derived from influenza vaccine in mice. Particularly, an induction of neutralizing antibody and memory B-cell against influenza, an increase in CD4 and CD8 T-cell proliferation, and enhanced levels of IL-2, IFN-γ, IL-5, IL-6 were highlighted [121].

Advax also enhanced the immunogenicity of hepatitis B surface antigen (HBs) in mice and guinea pigs, by increasing both anti-HBs antibody titers and anti-HBs CD4 and CD8 T-cells. Th1, Th2 and Th17 responses were increased too [122]. Astragalus polysaccharides were also used as adjuvants of Hepatitis B virus DNA vaccine in a mice model, showing increased HBsAg-specific antibody levels, higher activity of T cells, the production of IL-4, IL-2 and IFN-γ by CD4+ T cells, and IFN-γ expression of CD8+ T cells. Moreover, a stimulation of cytotoxic lymphocytes and dendritic cells maturation, and a reduction in the frequency of regulatory T cells were observed [123]. Mannans and fructooligosaccharide have also been shown to possess adjuvanticity [124,125].

Furthermore, indirect effects are involved in the immunomodulatory properties of polysaccharides. In particular, dietary fibers (e.g., inulin, mannan, β-glucan, pectin) are metabolized by intestinal bacteria in the anaerobic environment of the cecum and colon, so generating SCFA, such as acetate, propionate and butyrate [126]. These molecules are able to cross the gut epithelium and interact with surface receptors on the immune cells, such as the G-protein coupled receptors (GPRs) 41 and 43 [127]. The activation of GPRs by SCFA modulates inflammatory signalling pathways, such as NF-κB, ERK and p38 MAPK [128,129].

Moreover, it has been highlighted that SCFA can reach T lymphocyte nucleus, so modulating several functions through a histone deacetylase (HDAC) inhibition. Recently, SCFA have been reported able to induce T cells metabolic alterations by enhancing the mTOR complex activity. Particularly, after absorption into T cells, SCFA can stimulate the activity of mTOR complex, so increasing the conversion of pyruvate into acetyl-CoA. Moreover, the acetyl groups from SCFA can be link to CoA and enter the tricarboxylic acid cycle. The increased levels of citrate are exported from mitochondria into the cytoplasm, where the enzyme ATP citrate lyase converts it into acetyl-CoA, then used by histone acetyltransferases (HATs) for histone acetylation and the regulation of cytokine gene expression [126].

### 2.4. Clinical Studies

Some clinical studies have been carried out on the potential immunomodulatory properties of polysaccharides, and *A. membranaceus*, *E. purpurea* and β-glucan have been most investigated.

In a clinical trial on *A. membranaceus* by Jiang et al. [130], twenthy-eight stable continuous ambulatory peritoneal dialysis patients were treated with peritoneal dialysis fluid containing astragalus (20 mL/2 L) for one week. An increase in the macrophage phagocytic capacity, NO and TNF-α contents were observed in patients compared to those before the treatment [130]. Furthermore, Ji et al. [131] investigated the effect of astragalus pre-operative treatment of colorectal cancer patients (*n* = 128) on immune function. Results showed that astragalus pre-operative treatment promoted the NK cell activity in postoperative patients. In addition, the possible immunomodulatory activity of astragalus in patients with acute exacerbations of bronchial asthma (n = 72) has been investigated [132]. Particularly, it was observed that the combination of conventional therapy with astragalus injection for 14 days improved the effects of routine treatment, by enhancing T lymphocyte and NK-cells immune function.

Results of clinical trials on the immune system modulation by *E. purpurea* are controversial. Particularly, a randomized blinded trial carried out on 108 patients revealed that there was no significant difference in the incidence and severity of colds and respiratory infection between echinacea treatment (8 weeks) and placebo groups. However, a small decrease of total lymphocyte counts was observed [133]. 

Another randomized, placebo controlled, double-blind clinical trial investigated the effect of different echinacea preparations, namely Echinaforce^®^ (*E. purpurea* preparation from 95% herba and 5% radix), *E. purpurea* concentrate (same preparation at 7 times higher concentration), special *E. purpurea* radix preparation (totally different from that of Echinaforce^®^) on the reduction of the complaint index, defined by 12 symptoms in healthy, adult volunteers who caught a common cold. The treatment continued until the enrolled patients felt healthy again, but not longer than 7 days. The supplementation with Echinaforce^®^ and its concentrated preparation showed to be significantly more effective than the special echinacea extract or placebo. Moreover, all treatments were well tolerated [134]. Furthermore, prevention trials have been carried out, showing that echinacea products slightly reduce the risk of getting a cold in healthy individuals [135]. However, the heterogeneity (e.g., different species and part used) of preparations used in the trials makes the conclusions on the potential immunomodulatory properties of echinacea difficult.

Clinical trials concerning the β-glucan immunomodulatory properties have also been carried out, although in some cases, yeast-derived-glucan were used. Particularly, three randomized, double-blind, placebo-controlled studies have evaluated the effects of short-term β-glucan supplementation on children with chronic respiratory problems. After 30 days’ treatment, significant improvements in immunoglobulin, lysozyme, exhaled nitric oxide, and calprotectin production were found [136,137,138]. Furthermore, the combination of resveratrol plus carboxymethyl-β-glucan as a solution for aerosol has been tested in clinical trials. Particularly, the ability of the combination to prevent or treat recurrent respiratory infections in children was studied [139,140]. In both cases, resveratrol plus carboxymethyl-β-glucan had a positive impact on children clinical conditions. Indeed, nasal obstruction, rhinorrhea, sneezing, cough, fever, medication use, medical visits, and school absence were significantly reduced. Moreover, resveratrol plus carboxymethyl-β-glucan have also been shown to relief nasal symptoms in children with allergic rhinitis, due to pollen allergy [141]. 

At last, mannans should be mentioned. They have been reported to possess adjuvant-vaccine properties in clinical studies, probably mediated by its interaction with mannose receptors. Particularly, it has been shown that oxidized mannan-mucin 1 can be useful as an adjuvant in the breast cancer immunotherapy. Indeed, a 12–15 years follow-up has highlighted that it decreases the cancer recurrence rate and prolongs recurrence time, without inducing toxicity or adverse reactions [124]. 

## 3. Fatty Acids

### 3.1. Chemical Features

Fatty acids (FA) are a large group of lipids, characterized by a different number of carbons, arranged in a linear carbon chain skeleton of variable length with a terminal carboxylic group [142]. Based on the number of carbons in the chain, fatty acids can be classified as shortchain fatty acids (SCFA; aliphatic tails up to a maximum of six carbons), medium-chain fatty acids (MCFA; aliphatic tails of 7–12 carbons), long-chain fatty acids (LCFA; aliphatic tails of 13 to 21 carbons) and very long-chain fatty acids (VLCFA; aliphatic tails of 22 and more carbons) [143].

Among them, SCFA, such as acetate, propionate, and butyrate, are produced by gut microbiota enzymes (i.e., propionate-CoA transferase and propionaldehyde dehydratase) during the metabolism of carbohydrates and peptides containing branched-chain amino acids [144]. *Bacteroidetes* are reported to be mainly responsible for the production of acetate and propionate, while *Firmicutes* are the primary contributors of butyrate; however, other bacteria such as *Lactobacillus* and *Bifidobacterium* spp. are involved too [144]. 

Based on the presence of different double bonds in this structure, fatty acids can be distinguished in saturated fatty acids (SFA), lacking double bonds in their carbon backbone, and unsaturated FA (UFA), which may contain one or more double bonds, thus leading to monounsaturated (MUFA) and polyunsaturated FA (PUFA) [143]. SFA include palmitic acid (C16:0), lauric acid (C12:0), myristic acid (C14:0), and stearic acid (C18:0), whereas n-9 oleic acid (C18:1) is an example of MUFA. 

Furthermore, PUFA class includes fatty acids such as α-linolenic acid (ALA; C18:3), linoleic acid (LA; C18:2) and further long-chain metabolites [143]. The number of carbon atoms and unsaturated bond position are used for the systematic nomenclature of FA. Moreover, the greek letters omega (ω) and delta (∆) are included, to indicate how far a double bond is from the terminal methyl carbon and the presence and position of one or more double or triple bonds in the carbon backbone, respectively [143]. A further “ω” or “n” classification designates the position of the first double bond in the skeleton from the end opposite to the carboxy group. Accordingly, oleic acid is classified as a ω-9 (or n-9) fatty acid, while linoleic acid and *α*-linolenic acid are ω-6 (or n-6) and ω-3 (or n-3) fatty acids, as they contain the double bond nine, six and three carbons from the methyl end [143]. Nomenclature of the major representative fatty acids in the different FA classes is displayed in Table 3.

Unsaturated fatty acids can be characterized on the basis of the *cis-* or *trans-* orientation of the double bonds. Usually, natural fatty acids carry a *cis-* configuration, although some *trans-*fatty acids can also occur in foods as a consequence of the hydrogenation process, which can move double bonds from their naturally occurring position to a *trans-*configuration [143]. Trans-fatty acids are considered undesirable compounds in foods, as their intake is associated with an increased risk of cardiovascular and metabolic diseases [145]. 

PUFA can be further classified depending on the relative positions of the double bonds, as conjugated (double-bonded carbon atoms alternate with single bonds) and unconjugated (double bonds separated by one or more single bonds) [143]. Unconjugated PUFA, especially ω-3, ω-6, and ω-9 series, are the most occurring in nature. The most common conjugated PUFA are trienes, such as octadecatrienoic acids (e.g., punic acid, calendic acid). 

**Figure 6:**
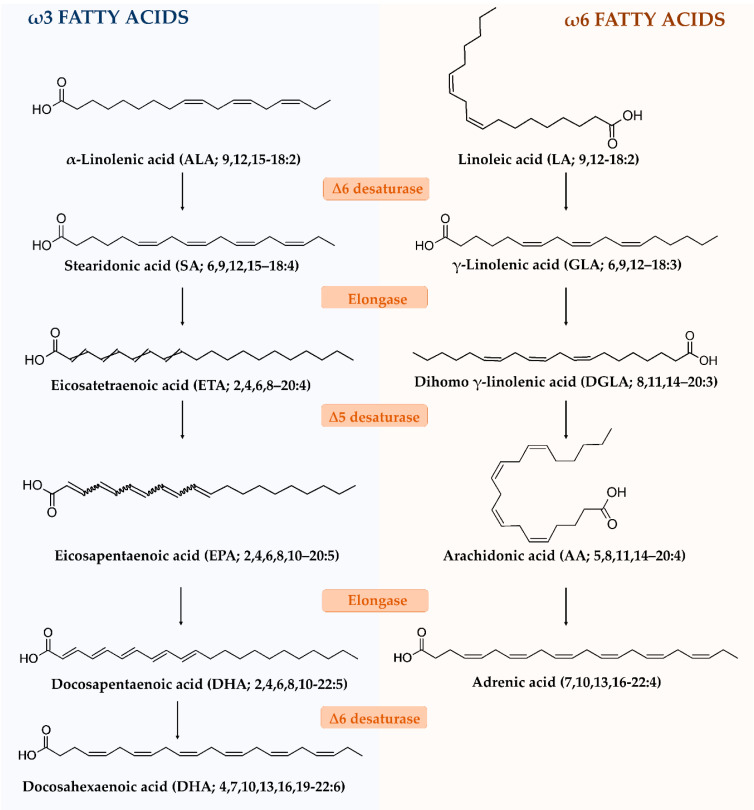
Biosynthetic pathways of ω-3 and ω-6 fatty acids.

Fatty acids within the series are biosynthetically related, being synthesized through enzymatic processes of desaturation, chain elongation, and chain shortening [146]. Particularly, the biosynthesis of ω-3 and ω-6 PUFA starts from α-linolenic acid (ALA or linolenate; 9,12,15–18:2) and linoleic acid (LA or linoleate; 9,12–18:2), respectively (Figure 6). 

These precursors cannot be synthetized by mammals, which lack the ∆12 and ∆15 desaturases responsible for the convertion of 18:1 ω-9 FA to 18:2 ω-6 and 18:3 ω-3 PUFA, and must be supplied by the diet, thus being considered as essential fatty acids. 

The initial rate-limiting step for the biosynthesis of ω3 and ω6 fatty acids is the insertion of a further double bond at the ∆6 carbon into the carbon chain of ALA and LA, through the help of a ∆6 desaturase enzyme: stearidonic acid (SA; 6,9,12,15–18:4) and γ-linolenic acid (GLA; 6,9,12–18:3) are formed, respectively. These compounds are converted to eicosatetraenoic acid (ETA; 2,4,6,8–20:4) and dihomo γ-linolenic acid (DGLA; 8,11,14–20:3) by the elongase 5, being further converted to eicosapentaenoic acid (EPA; 2,4,6,8,10–20:5) and arachidonic acid (AA; 5,8,11,14–20:4), by the addition of a double bond at the ∆5 position, through a ∆5 desaturase. Further elongations convert EPA and AA to docosapentaenoic acid (2,4,6,8,10–22:5) and adrenic acid (7,10,13,16–22:4); then, a desaturation by ∆6 desaturase generates docosahexaenoic acid (DHA; 4,7,10,13,16,19–22:6) [146]. Both series of fatty acids can be further metabolized by cyclooxygenase and lipoxygenase enzymes, to obtain eicosanoids, including prostaglandins, thromboxanes and leukotrienes, acting as central modulators of the inflammatory process [146]. 

The byosynthetic pathways of ω3 and ω6 fatty acids are interconnected; indeed, it is known that long-chain derivatives from linolenic acid are accumulated in tissue only slightly when competing ω6 analogues exceed their amounts. Therefore, suitable levels can be reasonably obtained through diet and when an optimum ratio of ω3 and ω6 series is maintained. 

### 3.2. Natural Occurrence

Fatty acids occur widely in nature, being identified in both animal tissue and plants. Particularly, short-chain saturated acids are components of milk fats: in bovine milk, butanoic acid along with other SCFA and MCFA have been reported [147]. Likewise, the MCFA lauric acid and myristic acid are the major components of the oils obtained from some Lauraceae and Myristiceae species [148]. Moreover, palmitic acid is the most representative SFA in vegetable oils, such as palm oil [149]. 

Fatty acids with immune modulating properties mainly belong to the long-chain classes; among them, punicic acid is a peculiar conjugated triene, found to be a unique component of pomegranate seed oil [150], while oleic acid is one of the most widely distributed fatty acids: it represents 49% to 83% of total FA in olive oil, although it does occur in high amounts in other oils, such as those from grape seeds, canola and sufflower [151,152]. 

Moreover, ω3 and ω6 fatty acids have been highlighted in several natural sources, wherein both series co-occur (Table 4), although in different amounts [153]. 

Some vegetable oils, including rapseed, hemp seed, and sunflower oils, contain higher levels of LA (essential ω6 PUFA), while ALA (essential ω3 PUFA) is in lower proportion; a similar trend has also been reported for soybean, corn, and for dried black walnuts and brazilnuts; conversely, higher amounts of ALA respect to LA are reported in flaxseed oil and in the seeds of chia and perilla [153]. Likewise, green leafy vegetables seem to be an interesting source of ALA [163]. Fish oils are also sources of both EPA and DHA (ω3 PUFA), with lower amounts of DPA (ω6 PUFA) [153]. 

Wild marine species showed to contain higher ω3 PUFA levels compared to farmed ones, likely due to the feed composition [153]. Some vegetables can supply both the essential PUFA and some derivative fatty acids. Particularly, the oils obtained from the seeds of *Borago* spp., *Echium* spp., *Ranunculus* spp. and *Oenothera biennis* L. have been reported to contain high levels of both LA and γ-linolenic acid (GLA) [159,160].

### 3.3. Pharmacological Properties: Preclinical Evidence

Fatty acids play energetic, metabolic, and structural functions, being the main component of phospholipids, triglycerides, diglycerides and monoglycerides. A separate category is represented by SCFA, which act as metabolites of carbohydrates, produced by gut microbiota: their role in the modulation of immune function is described in Section 2.3.

Long-chain fatty acids have been found involved in immune modulation, being able to affect both innate and adaptive response. Although specific profiles characterize each class of fatty acids, these effects are mainly ascribed to their ability to target the cell membrane, where they can be incorporated, thus changing membrane composition and fluidity and modulating membrane-protein interaction and signal transduction. Furthermore, a role in the control of inflammation has been reported. Epithelial growth factor receptors (a critical crossroad of multiple receptor pathways which is potentially implicated in the regulation of proliferation and possibly involved in atherogenesis) are considered possible targets for unsaturated fatty acids [164].

MUFA, especially oleic acid, have attracted great attention in the years as possible immunomodulating nutrients. Preclinical studies demonstrated the ability of oleic acid to modulate the immune system, through affecting both innate and adaptive immunity response [164]. Indeed, it diminished NK cell activity [165] and the expression of the leucocyte adhesion molecules, which have shown to be implicated in some pathophysiological conditions, such as rheumatoid arthritis [165]. Furthermore, it enhanced neutrophil aggregation and neutrophil-endothelial cell attachment, phagocytic and candidacidal capacities [166,167]. 

In regard to adaptive response, it inhibited the proliferation of immune cells, such as Jurkat T cells and lymphocytes, likely through the regulation of the cell cycle, although the true mechanisms remain to be clarified [164]. Similar suppressive effects were also highlighted for its synthetic analogue minerval and confirmed in animal models [164,168]. Furthermore, the treatment with oleic acid and minerval induced proapoptotic effects in Jurkat (T lymphocyte) and Raji (B lymphocyte) cells, likely due to mitochondrial depolarization and ROS production [168,169,170]. 

Recently, oleate has been reported to be able to protect macrophages from palmitate-induced lipotoxicity; moreover, it has been associated with an increase in the regulatory phenotype of the myeloid MSC-2 suppressor cells and suppression of activated T cells [171,172]. In the skin, oleic acid, along with other unsaturated FA, seems to be incorporated into the lipid moiety of *Staphylococcus aureus* Lpp, inducing an immune response against the pathogen [173]. 

Regarding conjugated PUFA, punicic acid has been shown to improve the immune system development, stimulate the CD4+ and CD8+ lymphocyte-mediated immunity and increase the immune response against viruses [174]. These immune boosting effects are due to nuclear peroxisome proliferator-activated receptor (PPAR)γ- and δ-dependent mechanisms, as punicic acid is able to act as an agonist of these receptors; in support, the loss of PPARγ in immune cells impaired its effects [174,175]. Moreover, punicic acid inhibited the TNF-α-induced priming of ROS production by inhibiting the Ser345-p47phox phosphorylation and upstreaming kinase p38MAPK; likewise, it blocked the TNF-α-induced release of myeloperoxidase from neutrophils, and decreased neutrophil-activation and ROS/MPO-mediated tissue damage *in vivo* [176]. Antinflammatory properties were found to be related to the activation of PPARγ and the suppressed expression of inflammatory genes (encoding cytokines, chemokines, cyclooxygenase, NO synthase, and metalloproteinases) [150].

Immunomodulatory properties of ω-6 and ω-3 PUFA have been highlighted in different preclinical models and have been associated with their ability to modulate the inflammatory process [177]. These fatty acids share common biosynthetic enzymes which mediate the production of different series of eicosanoids, starting from typical precursors, including dihomo-γ-linoleic acid (DGLA), arachidonic acid (AA) and eicosapentaenoic acid (EPA). Among prostanoids, three types of prostaglandins (PG), including PG1, PG2 and PG3, can be obtained. PG1 is associated with beneficial effects and lower inflammation, thus being considered as an antinflammatory prostanoid; conversely, PG2 has opposite behaviour, increasing inflammation, vasoconstriction and blood clotting. PG3 acts through a mixture of functions and is able to reduce the PG2-mediated inflammation [177].

**Figure 7:**
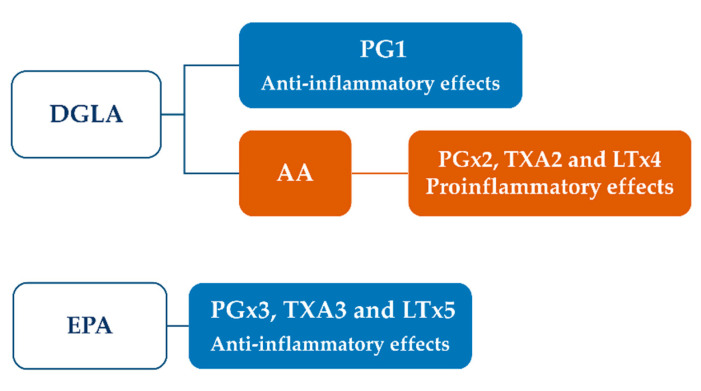
Role of ω-6 and ω -3 PUFA in the synthesis of proinflammatory or anti-inflammatory mediators.

Starting from DGLA, both anti-inflammatory PG1 and pro-inflammatory PG2, through the conversion into arachidonic acid, can be produced (Figure 7). 

This synthesis is controlled by the activity of Δ5-desaturase and Δ6-desaturase enzymes, which are often compromised during inflammatory conditions and diseases. It has been found that diets enriched in ω-3 fatty acids are able to activate the conversion of DGLA into PG1, whereas low ω-3 intake induces the conversion in AA with the synthesis of proinflammatory prostanoids [178].

AA can also be released by the cell membranes through the action of phospholipase A2 during cell injuries or changes in biomembrane composition, thus representing a physiological activator of inflammation as a defence response. AA and eicosapentaenoic acid (EPA) compete for the synthesis of different series of PG, mediated by cyclooxigenase, while 5-lipooxygenase (LOX) is involved in their conversion into thromboxanes and leukotrienes. Particularly, tromboxane A2 and leukotriene B4 are produced from AA, while tromboxane A3 and leukotriene B5 from EPA. EPA and DHA are also precursors of lipoxins, resolvins and protectins, which produced anti-inflammatory effects and regulate vascular tone and blood pressure [178]. Like punicic acid, the anti-inflammatory effects of EPA and DHA are mediated by the activation of the PPARα/γ [179]. 

Despite the antinflammatory role of ω-3 and ω-6-based diets, AA increases the plasmatic levels of proinflammatory eicosanoids, associated with an increased incidence of allergic and inflammatory disorders and with excessive cell proliferation. 

Anyhow, it is not clear the usefulness to select ω-3 with respect to ω-6 in the diet: a balance between ω-3 and ω-6 PUFA seems to be essential for mantaining ahealth status.

The ability of fatty acids to be incorporated in the cell membrane seems to represent a key mechanism accounting for the immunomodulating properties of ω-3 and ω-6 PUFA. Indeed, immune cells (i.e., T cells and neutrophils) can incorporate exogenous fatty acids into membrane with a lateration in the function of cell surface pattern recognition receptors [180]. 

Dietary ω-3 PUFA has been shown able to modulate the macrophage function, through the activation of G protein coupled receptors (GPR) and to induce a shift to an anti-inflammatory phenotype [49]. Modulating signalings through the GPR receptor activation can also affect leukocyte function. Likewise, an inhibition of the pro-inflammatory phenotype of dendritic cells and of the T cell responses has been reported [49]. They are also able to inhibit neutrophil and monocyte adhesion, depending on the activation of PPAR-*α* [49]. 

Conversely, ω-6 PUFAs seem to promote inflammation, associated with incresead ROS levels, in neutrophils [49]. Particularly, linoleic acid increased the marginated pool of neutrophils in tissues by the induced expression of adhesion molecules; it also complexed with the anti-inflammatory molecule 1-antitrypsin, thus reducing LPS-induced IL-1 secretion in neutrophils [49]. On the whole, preclinical evidence highlighted that these fatty acids could increase neutrophil function, thus promoting innate immunity. 

Regarding adaptive immunity, ω-3 PUFA have been reported able to improve the mitogen-mediated activation of immune cells and to promote the development of a TH2-type immune response [180]. Moreover, an increased production of associated anti-inflammatory cytokines like IL-4, in spite of a reduction of pro-inflammatory TNF-α, was found [180]. Similar effects were highlighted with both fish oil-enriched diets and the purified EPA and DHA [49].

The beneficial influence of ω-3 PUFA has been highlighted also on epithelial cells during inflammation, being able to restore impaired barrier function and reduce the production of pro-inflammatory mediators [49]. Moreover, a strictly interplay between omega-3 fatty acids, immunity and gut microbiota has been reported and seems to be an essential factor to maintain the intestinal wall integrity. These effects have been ascribed to the ability of ω-3 PUFA to positively affect the microbiota composition and increase the production of anti-inflammatory compounds, like short-chain fatty acids [181]. 

Although a major interest over the years has been focused on marine sources of ω-3-enriched oils or on pure compounds, some plant species have been studied for their immunomodulating and anti-inflammatory properties, likely ascribable to ω-3 and/or ω-6 PUFA, although the major evidence has been highlighted for *Linum usitatissimun* L., *Oenothera biennis* L. and *Borago officinalis* L. [160,182,183].

The seed oil from *L. usitatissimum*, also known as flaxseed oil, has been reported to induce immunomodulating effects, likely through suppressing cell mediated immunity, without the involvement of humoral immunity. Being a rich source of ALA, its effects are mainly ascribed to this compound, although further studies suggested a possible contribution of bioactive phenolics [184]. Flaxseed oil was found to be effective in reducing skin inflammatory responses, although with a lower immunosuppressive power with respect to fish oil [185]. Moreover, it improved systemic and gut immunity, in a piglet model with intrauterine growth retardation: increased plasma concentration of immunoglobulin G, decreased CD3+CD8+ T lymphocytes, and the downregulation of genes expression for proinflammatory factors have been reported [186].

Regarding *O. biennis*, the administration of the seed oil (namely, evening primrose oil) in animal models enhanced PGE1 synthesis in peritoneal macrophages, decreased PGE2 amounts in granulocytes, and suppressed the natural killer (NK) cell activity and lymphocyte proliferation; moreover, it decreased the serum levels of interferon γ (IFN-γ) and MCP-1, while stimulating TNF-α [187,188,189,190,191]. Furthermore, anti-inflammatory effects have been found to be involved in the immunomodulation by evening primrose oil [160]. 

These effects were ascribed to the content of GLA, whose T-regulatory cell activity in autoimmune disease models was highlighted [192]. However, a contribution of LA to the antinflammatory effects seems to be likely; indeed, LA can itself modulate inflammation as it is metabolized by LOX to hydroxyoctadecadienoic acids (HODEs) and oxo-HODEs, characterized by antinflammatory properties [193]. 

Similarly, the seed oil from *B. officinalis* seeds produced immonomodulating and antinflammatory effects, likely through its GLA content [183]. A chemotactic migration of monocytes to necrotic site that differentiate into macrophages is associated with the administration of this product. Moreover, it is known to reduce the levels of proinflammatory cytokines, such as TNF-α, and to promote PGE1 generation; a reduced expression of inflammatory genes, especially those of macrophages involved in atherosclerosis, has been reported too [183,184,185,186,187,188,189,190,191,192,193,194]. 

### 3.4. Clinical Studies

Clinical studies mainly focused on the effects of fatty acid-enriched diet on inflammation, although specific immune-based pathological conditions associated with inflammation were assessed too. Regarding MUFA, few studies are available, and results differ from those in animal models. Indeed, a MUFA-rich diet (with highly refined olive oil for 8 weeks) did not alter the immune function in healthy subjects; such effects could be due to the high amounts administered in animal models [164]. Conversely, clinical evidence about the immunomodulatory power of punicic acid in healthy or sick subjects is lacking [150].

The ω-3 PUFA series and the relative enriched fish oils have been mainly evaluated for their immunomodulating and antinflammatory effects in humans. Although preclinical evidence highlighted their ability to influence both innate and adaptive immunity, the clinical relevance of these results remains to be clarified, due to lacking or inconclusive data [49]. 

Inadequacy of clinical results should be due to the different doses used in preclinical studies, wherein often high fatty acids levels were administered; moreover, other factors such as genetic and epigenetic heterogeneity of the recruited subjects, diet diversity, nutritional habits and microbiome can be considered as additional confounding factors [49]. 

Although limitations of clinical studies require further confirmation, ω-3 PUFA intake produced significant clinical benefits and reduction of the symptoms in patients with autoimmune disorders, especially rheumatic diseases and systemic lupus erythematosus [195,196,197]. In support, low levels of PUFAs have been found in the serum of patients with rheumatic diseases [198]. Conversely, inconsistent results are reported for multiple sclerosis, thus the possible usefulness of these fatty acids as supportive therapy requires more clinical trials [199]. 

Regarding ω-6 PUFA, although they are associated with possible increased inflammatory conditions, being arachidonic acid a presursor of proinflammatory prostanoids, such a risk is not confirmed by clinical evidence [200]. Indeed, studies in healthy human adults highlighted that an increased intake of these fatty acids did not induce inflammation; conversely, epidemiological evidence reported reduced inflammatory conditions [200]. The antinflammatory effects of LA have been reported too [193]. Similarly, increased LA intake was found to be not related to increased amounts of ARA and proinflammatory factors; however, an inverse correlation with EPA and DHA was reported [201]. This suggests that the interaction between ω-e and ω-6 series is regulated by complex mechanisms that requires further clarifications.

Major clinical studies have been performed using evening primrose oil (from the seeds of *O. biennis*), as a source of LA and GLA, in inflammatory diseases associated with immune system disorders, including atopic dermatitis, psoriasis, multiple sclerosis and rheumatoid arthritis. Standardized oils for the content in LA and ALA (for instance, Efamol is titred to contain 72% LA and 9% GLA) were usually used [202]. 

The treatment with evening primrose oil (4 and 7 weeks) produced clinical improvements in patients with atopic dermatitis, as revealed by measuring the SCORing atopic dermatitis [203]. Some beneficial effects were also reported in multiple schlerosis patients, although the few available studies limited the evidence in this disorder. 

Conversely, evening primrose oil in combination with fish oil and vitamin E (Efamol Marine) failed to improve the symptoms of psoriasiac patients but produced antinflammatory effects [204]. Similarly, in association with ω-3 fatty acids, it did not induce improvements in patients with rheumatoid arthritis [205]. A Cochrane revision highlighted moderate evidence for oils containing GLA (i.e., evening primrose, borage, or blackcurrant seed oil) to produce benefit in rheumatoid arthritis [205,206]. Evening primrose oil along with borage oil were not effective to treat eczema too [207]. Highly variable results were also obtained for borage oil in the treatment of atopic dermatitis, although, in all the studies, a moderate efficacy degree was displayed [208]. 

Regarding flaxseed oils, some clinical trials higlighted a significant improvement of inflammatory parameters in subjects with cardiovascular diseases non-associated with the immune system [209].

Reported studies, although performed in pathological conditions associated with immune system disfunction, did not give a direct measure of the immunomodulatory effects of the treatments. Furthermore, specific and high-quality studies are required for better characterizing the possible usefuleness of these PUFA-enriched oils as anti-inflammatory and immunomodulating treatments.

## 4. Labdane Diterpenes

### 4.1. Chemical Features

**Figure 8:**
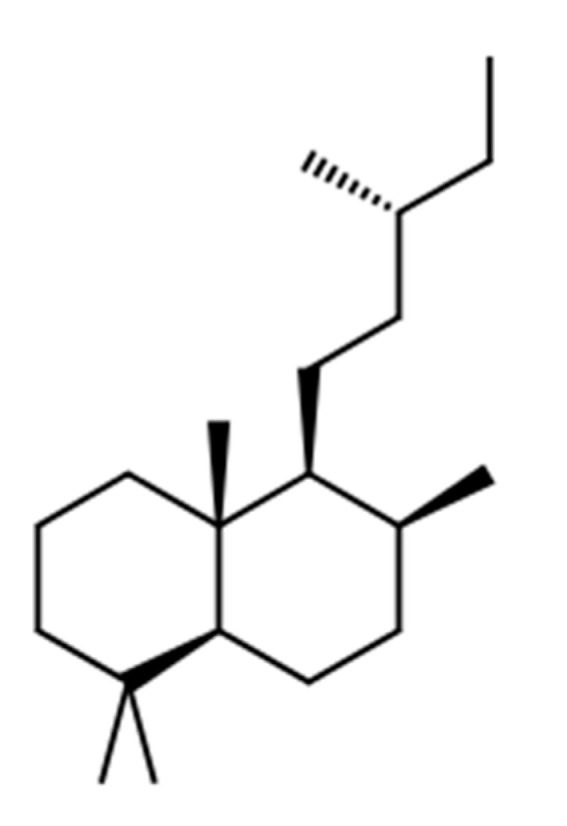
Chemical structure of the skeleton of labdane compound. The figure has been obtained using the ChemSpider^®^ chemical structure database.

Labdane compounds have a molecular formula C_20_H_38_ with an average mass of 278.516 Da (Figure 8). Labdane-related molecules have a hydrocarbon skeleton, originated from dual biosynthetic cyclization and/or rearrangement reactions, produced through the biosynthetic pathway of gibberellin phytohormones by the diterpene cyclases. The labane diterpenoids belong to a superfamily of natural products, in which the hydrocarbon skeleton might serve as privileged scaffolds for their biological activity [210].

### 4.2. Natural Occurrence

Labdane diterpenes have been found in the various matrix of vegetal origin (leaves, rizomes, fruits, etc.) of different plants. In Table 1, some of them (where diterpenes have been found), their botanical family (in parentheses) and the part of plant of biological interest are reported. 

**Figure 9:**
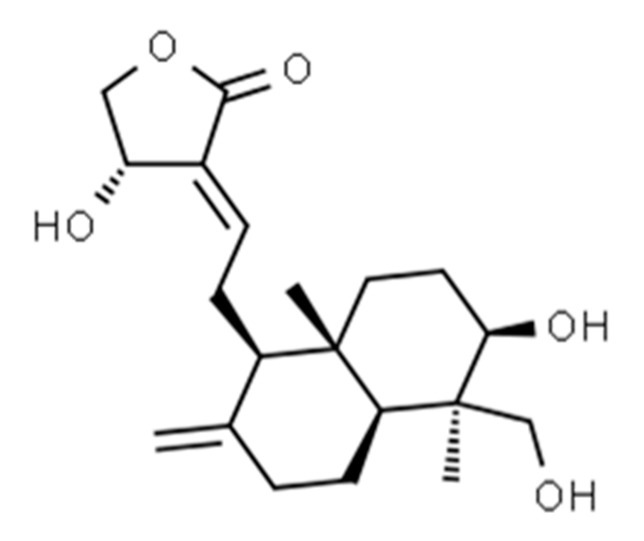
Chemical structure of andrographolide. This has been obtained using ChemSpider^®^ chemical structure database.

Some labdane diterpenoids, isolated from plant matrix, include the following: andrographolide (Figure 9) (from *Andrographis paniculata* (Burm.f.) Nees) [211], labda-8(17), 12-diene-15, 16-dial (from *Curcuma amada* Roxb) [212], podoimbricatin C (a 12,17-cyclo-labdane diterpenoid from *Dacrycarpus imbricatus* (Blume) de Laub) [213] chapecoderins A-C (from *Echinodorus macrophyllus* (Kunth) Micheli), [214], (4R,5S,9S,10R)-13-des-ethyl-13-oxolabda-8(17),11E-dien-19-oic acid (from *Juniperus oblonga* M. Bieb) [215], leoheteronin D and leojaponin A (from *Leonurus japonicus* Houtt) [216], marrubasch A-F and marrubenol (from *Marrubium aschersonii* P.Magnus), marrulibanoside (from *Marrubium globosum* Boiss. and Balansa) [217], vitexlimolides A–C (from *Vitex limonifolia* Wall. ex C.B. Clarke) [218].

### 4.3. Pharmacological Properties: Preclinical Studies

The body’s defense responses can be improved through various properties induced by plants. Some of them, referring to the plants in Table 5, are shown below. For each plants and plant-derived nutraceutical, only properties potentially attributable to the labdane skeleton and useful to improve the immune system are reported. 

As both inflammation (biological response of body tissues to harmful stimuli) and oxidative stress (imbalance between reactive oxygen species and a biological system’s ability to detoxify/repair the resulting damage of the reactive intermediates) are the main self-defend methods to eliminate pathogens and protect living bodies, plants with antinflammatory and/or radical scavenger properties are considered too [211]. Indeed, labdane diterpenoids have recently gained greater attention from the scientific point of view, due to a wide range of biological activities, including the anti-inflammatory modulation of immune cell functions [217].

*A. paniculata* exhibited, *in vitro* and *in vivo*, various pharmacological activities, including antihyperglycemic, antiplatelet aggregation, anti-microbial, anti-inflammatory, anti-HIV, anti-cancer, anti-nociceptive activity, etc. It has also been used for autoimmune encephalomyelitis and, in Indian and Chinese medicine, for respiratory tract infections [217,219]. 

More recently, *A. paniculata* has been used to stimulate the immune system and treat myocardial ischemia [211]. *A. paniculata* inhibited interleukin (IL)-6, TNF-α mRNA, LPS-induced expression, and suppressed levels of TNF-α, IL-1β, JNK, C-reactive protein, and NF-κB [211]. Many labdane diterpenoids compounds have been found to act on the latter. The activation of the NF-κB pathway leads to several physiological responses, including inflammatory or innate immune response [217]. 

*In vitro*, andrographolide (the main phytoconstituent of *A. paniculata*) can inhibit inflammation, by regulating protein expression (cytokines, chemokines) and by reducing immune cell infiltration. Andrographolide was shown to inhibit also oxidative stress by binding to adenosine A2A receptor, by inducing nuclear factor (erthroid-derived 2)-like 2 (Nrf2) translocation, and by increasing the expression of superoxide dismutase, catalase, glutathione reductase, glutathione peroxidase-2 [211]. These effects can contribute to the immunoregulatory activity of this plant-derived nutraceutical, as it can modulate the innate and adaptive immune responses by regulating macrophage phenotypic polarization and antibody productions [25]. Moreover, it was found to exert cytotoxic/anticancer effects on almost all types of tumour cell lines (human leukemia, renal tubular epithelial cells, breast cancer cells, etc.), mainly by cell cycle arrest, autophagy, cell death, anti-inflammatory and immune system mediated effects [220].

Other preclinical studies have highlighted pharmacological properties of labdane diterpenoids-containing plants. Data are limited, and consequently also their preclinical evidence. Some examples are reported below.

*C. amada*, also known as mango ginger, and its labdane-diterpenoids have shown antiflammatory, antibacterial, insecticidal, antifungal, antipyretic, antioxidant, anticancer, and antitubercolar properties in preclinical trials [212].

*D. imbricatus* displayed cytotoxic and anti-neuroinflammatory activities, but it had no cytotoxic activity against human tumour cell lines [213].

*E. macrophyllus*, Brazilian plant, also known as “leather hat”, is used as a methanolic (which contains mainly labane diterpenoids, steroids, alcaloids, etc.) or aqueous extract (rich in flavonoids) of the aerial parts, leaves in particular. In folk medicine, *E. macrophyllus* is used for various illnesses (respiratoy and urinary diseases, rheumatoid arthritis, atherosclerosis, etc.), as it has been shown to possess tissues protective activity and immunosuppressive effects (impaired secretion and function of B and/or T cells), on humoral or cellular immune responses and on autoimmune rheumatic diseases [221]. 

In *in vitro/vivo* studies, the aqueous extract of *E. macrophyllus* exhibited strong antinflammatory activities by decreasing rats paw edema, inflammatory exudates, infiltrate tissues, NO production, LTB4 release, and neutrophil migration [222]. In preclinical studies, the methanolic extract of *E. macrophyllus* was not cytotoxic, genotoxic, mutagenic, and no acute toxicity (up to the maximum dose of 2000 mg/kg b.w.) has been observed in tested animals [221]. However, the extrapolation of animal experiments to clinical practice must be done with caution [223].

Compounds from *J. oblonga* have shown anti-tumor effects, through moderate cytotoxicity against human tumor cell lines obtained from various human tissues, including: hepatocellular carcinoma (HepG2), breast cancer (MCF-7), and cervical carcinoma cancer (HeLa) [215]. The berries from *J. oblonga* also have antimicrobial activity and anti-inflammatory effects. 

Labdane diterpenoid (e.g., leonurine), extracted from *L. japonicus*, exhibited cytotoxicity and cell cicle arrest against cancer cell lines and presented immunomodulatory and antinflammatory activities (suppresses TNF-*α*, NF-κB, and down-regulated expression of iNOS, COX2, and conseguently PEG2 and NO levels) [216].

*Marrubium* spp. (*aschersonii*, *globosum*, etc.) have multiple actions, including antimicrobial and anti-inflammatory activities. Marrubasch A-F and marrubenol, isolated from the ethanolic extract of *M. aschersonii*, exhibited weak reduction in iNOS activity and, consequentely, NO production [217]. Marrulibanoside, obtained from the aerial parts of *M. globosum*, inhibited catalytic activity of iNOS and COX-2enzymes, and consequentely, the PEG2 and NO production.

*V. limonifolia*, in preclinical trial, have shown a strong antiviral activity against coxsackievirus B3, human rhinovirus 1B, and enterovirus 71 (EV71). All of them could be responsible for various illnesses, ranging from common cold, hand, foot, and mouth diseases, to acute flaccid paralysis [218].

### 4.4. Clinical Studies

The clinical efficacy of the medicinal plants, and plant-derived nutraceuticals discussed above are almost totally lacking. Only for *A. paniculata* there are some evidence in humans. 

Andrographis extract (various and not standardized), andrographolide, and its derivatives have been studied in the treatment of various disease (multiple sclerosis, infection disease, gastro-intestinal upsets, respiratory ailments, pain), and in the maintenance of immune function. In this last context, it seemed to improve the response to cough and sore throat, shortening the sick leave/time to resolution [219]. The most interesting activity is the increase of CD4+ lymphocyte levels, in HIV-positive patients [211]. The increase in these lymphocytes testifies an improvement in the state of the immune system. Moreover, a Chinese product containing andrographolide improved the efficacy of glucocorticoids and immunoglobulin in patients with severe hand, food, and mouth disease. 

However, andrographolide is considered a hazard, as it is irritating, and its injectable use is limited because it could induce allergic reactions (erythema, pruritus, etc.), which are sometimes life-threatening [211]. 

Preclinical data suggested that andrographolide could be responsible of pharmacokinetics interactions, as it induced CYP1A2 [219]. The European Medicines Agency (EMA) reports a possibility of causing reproductive toxicity of Andrographis extracts (decreases in sperm motility and counts) [219]. On the other hand, no major adverse effects have been reported for *A. paniculata*; only minor side effects, mainly gastrointestinal, are known [219]. 

Notably, even if *A. paniculata* presents numerous pharmacological properties, andrographolide possess poor solubility (principally in DMSO), which severely limits the possibility of achieving a therapeutic effect (if not properly formulated). Its better absorption could be achieved by nano-formulations (e.g., nano-emulsion, nano-capsules).

## 5. Further Research

Immunomodulation by plant-based nutraceuticals represents an interesting tool to be exploited for the treatment and preventing purposes of immune system disorders, due to their multiple bioactivities, well tolerability and good patient compliance. However, as often reported for several herbal medicinal products, some points require being underlined to improve the research in the field and provide solid evidence to support their rational use. 

According to previous stated critical issues [224,225], herbal products under study must be characterized for the phytochemical composition, using validated analytical methodologies, and for the extraction procedures; moreover, the starting material should be fully defined in terms of origin (country and region), cultivation conditions, botanical identity and plant part. The content of specific compounds, used as analytical or active markers, should be determined too. These requirements are needed to ensure reproducible pharmacological/clinical activity and to compare different studies. Indeed, using nonstandardized phytocomplexes increases variability of the biological response, thus limiting the reliability and validity of the studies. 

Furthermore, to assess the pharmacological activity of specific compounds, purity (at least 95%) and identity should be characterized. Indeed, when assessed as mixtures, the subtle interactions which can be established among phytochemicals make it difficult to understand whether the observed benefits are attributable to a specific class or to the whole phytocomplex. For instance, both fatty acids and polyphenols can be involved in the immunomodulating effects of PUFA-enriched plant oils. Moreover, as found for both polysaccharides and fatty acids, among the same class, different subclasses can co-occur, thus contributing to the whole effects. 

Regarding preclinical studies, detailed methodologies, including information about specific extraction process, the choice of the tested concentrations and experimental procedures, vehicle effects, and comparison with standard effective compounds (positive controls) should be reported. 

In order to validate the “goodness” of the treatment, promising results in preclinical studies should be confirmed by clinical evidence of efficacy and lack of toxicological concerns for both the isolated compounds and the whole phytocomplex. At last, possible interactions with diet constituents or possible pharmacological treatments, as reported for andrographolide, which is a CYP1A2 inducer, should be considered.

As highlighted for a number of natural products, clinical evidence is a major challenge for plant-based immunomodulating nutraceuticals too, due to limited specific studies.

Moreover, methodological quality of the available trials was overall poor, the studies often being not blinded, protocol unavailable and lacking the standardization of tested products, thus making the claimed effect difficult to be reproduced. 

At last, standardized methodologies for systematic reviews and meta-analyses, such as the PRISMA guidelines [226], would allow a rational interpretation of the results and suggestions for future research.

## 6. Conclusions

Medicinal plants are rich sources of bioactive phytochemicals, characterized by multiple and often pleiotropic activities, which can be exploited both therapeutically and as nutraceutical strategies for preventive purposes. Among plant-based nutraceuticals, immunomodulators have been highlighted to be of interest as boosters of the immune system, to counteract infectious or exogenous injuries, immunosuppressor, to control the abnormal immune response occurring during autoimmune diseases, or as adjuvants, which contribute by modulating nonimmune targets. 

In this review, we highlighted the scientific evidence about the immunomodulating properties of three emerging classes of nutraceuticals, including polysaccharides, fatty acids and labdane diterpenes. Some of them, especially polysaccharides and labdane diterpenes, act as immune system booster, while fatty acids (MUFA and PUFA) mainly act as immunosuppressor, although punicic acid (a conjugate PUFA) exhibited immunostimulant properties. To date, these products are mainly used as well-tolerated food supplements, although the clinical evidence about their modulation of the immune functions is still limited. More investigations of better quality and specificity could strengthen the validity of using these plant-derived nutraceuticals in the immune system modulation.

## Figures and Tables

**Table 1:** Some examples of bioactive constituents obtained from immunomodulatory plants, and their effect on immune function.

Medicinal Plants	Phytochemicals	Effect on Immune Function/Type of Immunomodulation	References
*Acacia catechu* Willd. heartwood	Flavonoids, phenolic acids, catechins	Antinflammatory activity/Immunoadjuvant	[21]
*Aloe vera* (L.) Burm.f.	Acemannan, dihydrocoumarins	Adaptive immunity activation/Immunostimulant	[22,23,24]
*Andrographis paniculata* (Burm.f.) Nees	Diterpene lactones (andrographolide)	Modulation of innate and adaptive immunity/Immunosuppressor	[25]
*Artocarpus tonkinensis* A. Chev. Ex Gagnep	Auronol glycosides (maesopsin 4-O-glucoside and alphitonin-4-O-glucoside)	Inhibition of humoral and cellular adaptive immunity/Immunosuppressor	[26]
*Astragalus membranaceous* (Fisch.) Bge.	Polysaccharides	Activation of cellular immunity/Immunostimulant	[27]
*Boswellia serrata* Roxb. ex Colebr	Boswellic acid	Anti-anaphylactic and mast cell stabilization/Immunosuppressor	[28]
*Camellia sinensis* (L.) Kuntze	Polysaccharides	Activation of immunoreactivity through the modulation of gut microbiome/Immunostimulant effect	[20,29]
*Centella asiatica* (L.) Urban	Madecassoside	Regulation the abnormal humoral and cellular immunity/Immunosuppressor	[30]
*Curcuma longa* L.	Curcuminoids	IL-10-mediated anti-inflammatory and immunosuppressive activity/Immunosuppressor	[31]
*Echinacea purpurea* (L.) Moench	Alkylamides, glycoproteins, polysaccharides	Activation of cellular immunity and modulation of gut microbiome	[18]
*Glycyrrhiza glabra* L.	Triterpene saponins (glycyrrhizin)	Enhanced cellular immunity and antinflammatory activity/Immunostimulant	[32]
*Hypoxis rooperi* T. Moore	Phenolic glucosides (hypoxoside)	Antinflammatory properties/Immunoadjuvant	[33]
*Ocimum sanctum* L.	Monoterpenes (eugenol and methyleugenol), sesquiterpenes (β-caryophyllene)	Activation of innate and adaptive immunity/Immunostimulant	[34]
*Panax ginseng* C.A. Meyer	Triterpene saponins (ginsenosides)	Adaptogen effects, stimulation of immune systems via cytokine activation, modulation of gut microbiome	[19,35,36]
*Syzigium aromaticum* (L.) Merr. and L.M. Perry	Monoterpenes (eugenol), sesquiterpenes (β-caryophyllene)	Activation of adaptive humoral immunity/Immunostimulant	[37]
*Tinospora cordifolia* (Thunb.) Miers	Arabinogalactan	Activation of adaptive immunity/Immunostimulant	[38]
*Withania somnifera* (L.) Dunal	Steroidal lactones (withaferin A)	Adaptogen effects, stimulation of adaptive humoral and cellular immunity/Immunostimulant	[39,40]
*Zingiber officianalis* Roscoe	Phenolics (gingerols)	Activation of adaptive humoral immunity/Immunostimulant	[41]

**Table 2:** Examples of medicinal plants containing polysaccharides with immune system modulation activities.

Plants	Family	Plant Part of Biological Interest	References
*Aconitum carmichaeli* Debx.	Ranunculaceae	Roots	[76]
*Allium sativum* L.	Liliaceae	Bulb	[77]
*Aloe vera* L.	Liliaceae	Leaves	[78]
*Amorphallus konjac* Koch	Araceae	Tubers	[79]
*Anadenanthera colubrina* (Vell.) Brenan	Fabaceae	Gum	[80]
*Astragalus membranaceus* (Fisch.) Bge.	Fabacee	Root	[64]
*Carum carvi* L.	Apiaceae	Seeds	[81]
*Centella asiatica* L. Urb.	Apiaceae	Aerial parts	[82]
*Coffea arabica* L. and *C. robusta* L.	Rubiaceae	Beans	[83]
*Cyamopsis tetragonolobus* L.	Fabaceae	Seeds	[84]
*Dendrobium huoshanense* C.Z. Tang et S.J. Cheng	Orchidaceae	Stems	[85]
*Echinacea purpurea* L. (Moench)	Asteracee	Aerial parts	[86,87]
*Euterpe oleracea* Mart.	Arecaceae	Fruits	[88,89]
*Hordeum vulgare* var. Tyra	Poaceae	Stems	[90]
*Ipomoea batatas* L.	Convolvulaceae	Roots	[91]
*Juniperus scopolorum* Sarg.	Cupressaceae	Cones	[92]
*Lycium barbarian* L.	Solanaceae	Fruit	[93]
*Panax ginseng* C.A. Meyer	Araliaceae	Roots	[94]
*Picea abies* L.	Pinaceae	Softwoods	[95]
*Prunus dulcis* (Miller) D. A. Webb.	Rosacee	Seeds	[96]
*Sophora alopecuroides* L.	Fabaceae	Seeds	[97]
*Tanacetum vulgare* L.	Asteraceae	Florets	[98]
*Tinospora cordifolia* (Thunb.) Miers	Menispermaceae	Aerial parts	[99]
*Trigonella foenum-graecum* L.	Fabaceae	Seeds	[100]

**Table 3:** Nomenclature and chemical features of some representative long-chain fatty acids.

Fatty Acid	Class of Long-Chain Fatty Acid	IUPAC Name	Abbreviation	
			Delta (∆) Nomenclature	n- or ω-Nomenclature
Palmitic acid	SFA	Hexadecanoic acid	16:0	16:0
Stearic acid	SFA	Octadecanoic acid	18:0	18:0
Oleic acid	MUFA	(9E)-Octadec-9-enoic acid	18:1∆9	18:1n-9 or 18:1ω-9
Punicic acid	Conjugated PUFA	(9Z,11E,13Z)-9,11,13-Octadecatrienoic acid	18:3∆9,9,11,13	18:3n-5 or 18:3 ω-5
α-Linolenic acid (ALA)	PUFA	(9Z,12Z,15Z)-Octadeca-9,12,15-trienoic acid	18:3∆9,12,15	18:3n-3 or 18:3ω-3
Stearidonic acid (SA)	PUFA	(6Z,9Z,12Z,15Z)-Octadeca-6,9,12,15-tetraenoic acid	18:4∆6,9,12,15	18:4n-3 or 18:4ω-3
Eicosatetraenoic acid (ETA)	PUFA	(2Z,4Z,5Z,8Z)-2,4,6,8-Icosatetraenoic acid	20:4∆2,4,6,8	20:4n-3 or 20:4ω-3
Eicosapentaenoic acid (EPA)	PUFA	(2E,4E,6E,8E,10E)-2,4,6,8,10-Icosapentaenoic acid	20:5∆2,4,6,8,10	20:5n-3 or 20:5ω-3
Docosahexaenoic acid (DHA)	PUFA	(4Z,7Z,10Z,13Z,16Z,19Z)-Docosa-4,7,10,13,16,19-hexaenoic acid	22:6∆4,7,10,13,16,19	22:6n-3 or 20:6ω-3
Linoleic acid (LA)	PUFA	(9Z,12Z)-Octadeca-9,12-dienoic acid	18:2∆9,12	18:2n-6 or 18:2ω-6
γ-Linolenic acid (GLA)	PUFA	(6Z,9Z,12Z)-Octadeca-6,9,12-trienoic acid	18:3∆6,9,12	18:3n-6 or 18:3ω-6
Dihomo γ-Linolenic acid (DGLA)	PUFA	(8Z,11Z,14Z)-Icosa-8,11,14-trienoic acid	20:3∆8,11,14	20:3n-6 or 20:3ω-6
Arachidonic acid (AA)	PUFA	(5E,8E,11E,14E)-Icosa-5,8,11,14-tetraenoic acid	20:4∆5,8,11,14	20:4n-6 or 20:4ω-6
Docosapentaenoic acid (DPA)	PUFA	(7Z,10Z,13Z,16Z,19Z)-Docosa-7,10,13,16,19-pentaenoic acid	22:5∆7,10,13,16,19	22:5n-6 or 22:5ω-6

SFA, saturated fatty acids; MUFA, monounsaturated fatty acids; PUFA, polyunsaturated fatty acids.

**Table 4:** Major natural sources of long-chain monounsaturated (MUFA) and polyunsaturated fatty acids (PUFA) associated with immune system modulating activities and relative amounts.

	Species (Byproduct)	Amount (%)	References
*MUFA*			
Oleic acid	*Olea europea* L. (fruit oil)	56–84% ^a^	[151]
	*Vitis vinifera* L. (grapeseed oil)	9–17%	[154]
	*Brassica* spp. (rapeseed oil)	60–80%	[155]
*Conjugated PUFA*			
Punicic acid	*Punica granatum* L. (pomegranate seed oil)	70–85%	[150]
	*Momordica charantia* L. (bitter gourds seed oils)	1.5–16%	[156]
*Essential ω3-PUFA*			
α-Linolenic acid (ALA)	*Linum usitatissimum* L. (flaxseed oil)	55–57%	[153,157]
	*Salvia hispanica* L. (chia seeds)	17.8%	[153]
	*Perilla frutescens var. frutescens* L. (seeds)	54–64%	[158]
EPA, DHA	Fish oils	4–13%	[153]
*Essential ω6-PUFA*			
Linoleic acid (LA)	*Borago* sp. (seed oil)	26.8–37.9%	[159]
	*Oenothera biennis* L. (seed oil)	70–74%	[160]
	*Cannabis sativa* L. (hamp seed oil)	27–58%	[153,161]
	*Glycine max* (L.) Merr. (soybean)	50.4%	[153,161]
	*Bertholletia excelsa* (Dried brazilnuts)	23.8%	[153,161]
	*Juglans regia* L. (dried walnuts)	33.8%	[153,161]
	*Zea mays* L. (corn)	53.2%	[153,161]
γ-Linolenic acid (GLA)	*Borago* sp. (seed oil)	9.6–39.8%	[159]
	*Oenothera biennis* L. (seed oil)	8–10%	[160]
	*Ribes nigrum*	15–19% ^a^	[162]
	(seed oil)		
	*Echium* spp. (seed oil)	31.2–47.1%	[159]
	*Ranunculus* spp. (seed oil)	37.9–39.7%	[159]

^a^ referred to the total amount of fatty acids.

**Table 5:** Examples of medicinal plants containing labdane diterpenoids, with immune system modulation activities.

Plants	Family	Part of the Plant of Biological Interest	References
*Andrographis paniculata* (Burm.f.) Nees	Acanthaceae	Aerial parts, roots	[211,219]
*Curcuma amada* Roxb	Zingiberaceae	Rizome	[212]
*Dacricarpus imbricatus* (Blume) de Laub	Podocarpaceae	Bark	[213]
*Echinodorus macrophyllus* (Kunth) Micheli	Alismataceae	Leaves	[214]
*Juniperus oblonga* M. Bieb	Cupressaceae	Berries	[215]
*Leonurus japonicas* Houtt	Lamiaceae	Aerial parts	[216]
*Marrubium* L. Spp.	Lamiaceae	Aerial parts	[217]
*Vitex limonifolia* Wall. ex C.B.Clarke	Lamiaceae	Leaves	[218]
